# Alveolar Overdistension as a Cause of Lung Injury: Differences among Three Animal Species

**DOI:** 10.1100/2012/985923

**Published:** 2012-05-03

**Authors:** Manuel García-Delgado, Inés Navarrete-Sánchez, Virginia Chamorro-Marín, Juan Carlos Díaz-Monrové, Javier Esquivias, Enrique Fernández-Mondéjar

**Affiliations:** ^1^Intensive Care Unit, Hospital Universitario Virgen de las Nieves, 18014 Granada, Spain; ^2^Experimental Unit, Hospital Universitario Virgen de las Nieves, 18014 Granada, Spain; ^3^Emergency and Critical Care Department, Hospital SAS, 11407 Jerez, Spain; ^4^Pathology Department, Hospital Universitario San Cecilio, 18012 Granada, Spain

## Abstract

This study analyses characteristics of lung injuries produced by alveolar overdistension in three animal species. Mechanical ventilation at normal tidal volume (10 mL/Kg) and high tidal volume (50 mL/Kg) was applied for 30 min in each species. Data were gathered on wet/dry weight ratio, histological score, and area of alveolar collapse. Five out of six rabbits with high tidal volume developed tension pneumothorax, and the rabbit results were therefore not included in the histological analysis. Lungs from the pigs and rats showed minimal histological lesions. Pigs ventilated with high tidal volume had significantly greater oedema, higher neutrophil infiltration, and higher percentage area of alveolar collapse than rats ventilated with high tidal volume. We conclude that rabbits are not an appropriate species for in vivo studies of alveolar overdistension due to their fragility. Although some histological lesions are observed in pigs and rats, the lesions do not appear to be relevant.

## 1. Introduction

Mechanical ventilation-associated lung injury is a clinically accepted fact and considered one of the main factors for the worsening or persistence of acute lung injury. The mechanism by which alveolar overdistension causes lung injury is not fully understood and is probably multifactorial. A major role is played by disruption of the alveolar-capillary membrane [1], surfactant alteration [2, 3], and slowing of alveolar fluid reabsorption [4], among many other factors.

Lung injuries from alveolar overdistension have been reported by several experimental research groups, predominantly in small animals (rats) [5, 6]. This type of injury has also been reported in larger animals, such as pigs or sheep [7, 8], but there have been far fewer studies, and it is suspected that the intensity of injury may vary among species, with large animals being more resistant to overdistension manoeuvres than small ones [9]. This hypothesis has not been verified, but there is evidence that distinct animal species behave differently against diverse lung injuries [10], suggesting that the response to alveolar overdistension may also differ among animal species. Knowledge of interspecies variations may be important in explaining the different results observed in studies on different species. It may also help to establish the degree to which results can be extrapolated, not only from the experimental to clinical setting but also from one to another species.

The objective of this study was to determine the histological and morphometric alterations produced by intermittent alveolar overdistension manoeuvre in three animal species frequently used in experimental studies.

## 2. Material and Methods

The study was approved by the ethical committee of our hospital, and the animals were managed according to Spanish norms for the protection of experimental animals (Royal Decree 1201/2005).

The experiment was conducted on pigs, rabbits, and rats. Two different mechanical ventilation protocols, normal tidal volume (10 mL/Kg) and high volume (50 mL/Kg), were applied in each species, as detailed below. After 30 min of mechanical ventilation, animals were killed by injection with potassium chloride, and the lungs were extracted by sternotomy for histological, morphometric, and gravimetric analyses. The left bronchus was ligated, and this lung was separated for gravimetric analysis. The right lung was infused with a 10% formaldehyde solution to a pressure of 20 cm H_2_O and then kept in a 10% formaldehyde bath for at least 72 h for subsequent histological analysis. Both histological and morphometric analyses were conducted by a pathologist blinded to the tidal volume group allocation of the animals.

### 2.1. Experiments in Pigs

Twelve mixed-breed pigs weighing 24–32 Kg were premedicated with intramuscular (i.m.) injection of ketamine (10 mg/Kg) and azaperone (5 mg/Kg). Anaesthesia was induced with intravenous (i.v.) injections of atropine (1 mg), ketamine (2 mg/Kg), and fentanyl (0.15 mg), immediately followed by tracheotomy and intubation with a cuffed tube (6.5 mm internal diameter). Anaesthesia was maintained with continuous infusion of ketamine (20 mg/Kg/h) and atracurium (1 mg/Kg/h), administering supplementary boluses of fentanyl and atracurium when necessary. The animals received a continuous infusion of 0.9% saline solution (3 mL/Kg/h) throughout the experiment.

**Groups**

Pigs were assigned to a normal tidal volume group (10 mL/Kg), respiratory rate of 16 per min (*n* = 6) or high tidal volume group (50 mL/Kg), respiratory rate of 12 per min (*n* = 6). The I : E ratio (1 : 2) and FiO_2_ (100%) were the same for both groups.

### 2.2. Experiments in Rabbits

Twelve white New Zealand rabbits, weighing 2.1–3.1 Kg were premedicated with i.m. ketamine (10 mg/Kg). Anaesthesia was induced with i.v. injections of atropine (0.5 mg), ketamine (2 mg/Kg), and fentanyl (0.05 mg), immediately followed by tracheotomy and intubation with a cuffed tube (3 mm internal diameter). Anaesthesia was maintained with a continuous infusion of ketamine (20 mg/Kg/h) and atracurium (1 mg/Kg/h), administering supplementary boluses of fentanyl and atracurium when necessary. A 0.9% saline solution infusion of 3 mL/Kg/h was administered throughout the experiment.

**Groups**

Rabbits were assigned to a normal tidal volume group (10 mL/Kg), respiratory rate of 40 per min (*n* = 6) or high tidal volume group (50 mL/Kg), respiratory rate of 20 per min (*n* = 6). The I : E ratio (1 : 2) and FiO_2_ (100%) were the same for both groups.

### 2.3. Experiments in Rats

Twelve Wistar rats weighing 0.290–0.380 Kg were premedicated with inhaled ether. Anaesthesia was induced with intraperitoneal injection of ketamine (75 mg) and atropine (0.5 mg), immediately followed by tracheotomy and intubation with a 14 G catheter, administering supplementary boluses of ketamine when necessary.

**Groups**

Rats were assigned to a normal tidal volume group (10 mL/Kg), respiratory rate of 75 per min (*n* = 6) or high tidal volume group (50 mL/Kg), respiratory rate of 40 per min (*n* = 6). The I : E ratio (1 : 2) and FiO_2_ (100%) were the same for both groups.

### 2.4. Histological Analysis

In pigs and rabbits, three 1 × 1 cm blocks were obtained from the central and posterior part of each lobule of the right lung. In rats, a longitudinal section was obtained such that all lobules were represented. Haematoxylin-eosin-stained sections were examined by light microscopy, and a previously published histological score was applied [11] that assesses five parameters: oedema, neutrophil infiltration, haemorrhage, bronchial epithelial desquamation, and hyaline membrane formation. The severity was scored on a 5-point scale: 0 for no or very minor, 1 for modest and limited, 2 for intermediate, 3 for widespread or prominent, and 4 for widespread and most prominent. In pigs and rabbits, the mean score of the 18 extracted blocks was calculated to yield a global histological score.

### 2.5. Morphometric Analysis

 Morphometric analysis was conducted to evaluate nonexpanded or collapsed alveolar areas; 10 digitalised images were obtained from each histological section, and images were captured at 10x with a Sony CCD AVC-7 video camera (Sony Corporation, Tokyo, Japan) adapted to a Nikon Labophot-2A microscope (Nikon, Tokyo, Japan). Visilog 3.6 (Noesis, Velice, France) software was used to measure alveolar areas, constructing a routine to permit quantification of alveolar areas, considering areas sized <50 pixels to be those with a tendency to collapse.

### 2.6. Gravimetric Analysis

After separating the left lung of each animal, it was weighed and placed in an oven for ≥72 h at 80°C. After removal from the oven, it was reweighed for calculation of the wet/dry weight ratio.

### 2.7. Statistical Analysis

SPSS 15.0 (SPSS, Chicago, IL, USA) was used for the data analyses. Quantitative variables were expressed as mean and standard deviation and the Mann-Whitney test was used to compare means of variables with abnormal distribution. Qualitative variables were expressed as percentages (mean percentage ± standard deviation), and the chi-square test was used for comparisons. A *P* < 0.05 was considered significant.

## 3. Results

All pigs and rats survived in both normal and high tidal volume groups, and no cases of barotrauma were detected. In contrast, five of the six rabbits in the high tidal volume group developed tension pneumothorax and/or pneumoperitoneum, and three died after <30 min of mechanical ventilation. As a consequence, this experiment was halted and the results for rabbits were excluded from the following analyses.

### 3.1. Histological Analysis

**Figure 1:**
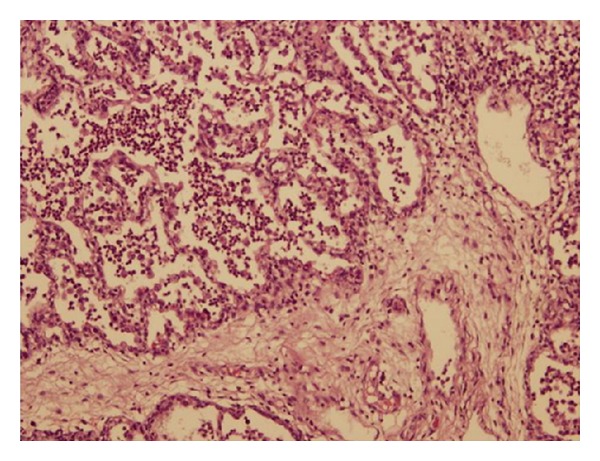
The image shows neutrophil infiltration in a pig with high tidal volume (P-HtV group).

Globally, the histological lesions were minimal, especially in the two rat groups. The total histological score was higher in pigs than in rats (Table 1), and the pig groups showed a more frequent presence of oedema and neutrophil infiltration (Figure 1). There was little formation of hyaline membrane, but this was significantly greater in the pigs ventilated with high versus normal tidal volumes (0.85 ± 0.69 versus 0.05 ± 0.13, *P* < 0.05).

### 3.2. Gravimetric Analysis (Wet/Dry Lung Weight)

The wet/dry weight ratio was significantly higher in the pigs treated with high versus normal tidal volume (5.60 ± 0.37 versus 4.33 ± 0.99, *P* < 0.01). In contrast, this ratio was significantly higher in the rats treated with normal versus high tidal volume (4.10 ± 0.08 versus 4.44 ± 0.1, *P* < 0.05) (Table 2).

### 3.3. Morphometric Analysis

The percentage area of alveolar collapse was significantly higher in pigs than in rats (Table 3).

In the pigs, the percentage area of collapse was higher (*P* < 0.05) in those treated with high (54.3 ± 8.4%) versus normal tidal volume (43.1 ± 10.5%). Comparing the percentage collapse areas of the upper, middle and lower lobes were 46.3%, 56.4%, and 60.1%, respectively, in the pigs with high tidal volume versus 34.5%, 45.8%, and 48.8%, in the normal tidal volume group; differences between the groups were significant for upper and lower lobes (*P* < 0.05) but not for middle lobes.

In the rats, the percentage area of collapse was higher (*P* < 0.05) in those treated with normal (38.5 ± 10.2%) versus high tidal volumes (26.8 ± 4.9%).

## 4. Discussion

Mechanical ventilation with high tidal volume (50 mL/Kg) for 30 min produced an increased tendency to alveolar collapse in intact pigs, but no other major histological lesions were detected. Almost no lesions were observed in rats after the same manoeuvre. In contrast, it caused early alveolar rupture with associated pneumothorax in rabbits, and their lower resistance to alveolar overdistension makes this species inappropriate for this type of experiment.

The vulnerability of rabbits to lung injuries has been previously reported. Studies on resistance of pulmonary capillaries, found that a much lower transmural pressure was needed to produce capillary stress failure in rabbits than in dogs or horses [12], which may be related to differences in capillary radius and blood-gas barrier thickness. In the present study, rabbits showed a much lower tolerance to alveolar overdistension in comparison to pigs or rats, which may be partly explained by the extreme thinness of their blood gas barrier. Other authors found a significant tendency to barotrauma and air leaks in rabbits [13, 14].

The same aggressive ventilation protocol produced different histological lesions between pigs and rats, with a predominance of oedema and neutrophil infiltration in the pigs and a tendency to haemorrhage in the rats. The gravimetric analysis also revealed differences. In the pigs, lungs were significantly heavier in the high versus normal tidal volume group, whereas in the rats, they were significantly heavier in the normal versus high tidal volume group. However, this surprising result in the rats may be a methodological artefact, since the blood pressure was not measured in this species, and alveolar overdistension in the high volume group may have led to hemodynamic compromise, lung hypoperfusion, and a smaller perfused lung vascular surface [15], hence reducing the formation of lung oedema, which primarily requires lung circulation. In contrast, arterial blood pressure was monitored in the pigs, and hemodynamic alterations were detected and immediately treated with volume infusion and inotropes when necessary, ensuring that a effective lung circulation was always maintained. These circumstances may have influenced the results, preventing conclusions from being drawn.

The most relevant finding was the higher tendency to collapse after ventilation with high tidal volume in the pigs (mostly in lower lobes) than in the rats. It has been reported that the increased alveolar surface area produced by ventilation with high tidal volumes produces surfactant inactivation. Thus, proteoglycan breakdown and glycosaminoglycan fragmentation contribute to an alteration of the extracellular matrix that may produce greater lung collapse [16].

In contrast to our results, other authors found that ventilation with high tidal volume for 30 min caused severe lung injuries in rats [5] and in dogs [7]; however, there are important methodological differences that may explain these discrepancies. In rat studies, the tidal volume applied is generally much higher than used in the present study, ventilating with a pressure of 45 cm H_2_O and generating a volume of around 90 mL/Kg, almost double the volume in the present study. In rats ventilated with lower volumes (peak pressure of 30 cm H_2_O), only a moderate interstitial oedema was detected after 60 min of ventilation [17], and other authors found no major histological lesions in rats ventilated with 25 mL/Kg for 30 min [18]. The tidal volumes used in studies that show overdistension lesions in larger animals are also much higher than in the present study. Parker et al. [19] observed an increase in microvascular permeability in dogs ventilated with peak pressures of 64 cm H_2_O and with a very high mean volume of around 90 mL/Kg. Other studies in large animals using similar volumes to those in our study required a much longer ventilation time to produce histological lesions or severe lung function impairment [20, 21].

We can conclude that animal species respond differently to high tidal volumes. Rabbits are not appropriate animals for this type of experiment, whereas pigs and rats show no important histological lesions after 30 min of ventilation.

## Figures and Tables

**Table 1:** Histological score.

	Pig groups		Rat groups	
	P-NtV	P-HtV	Rat-NtV	Rat-HtV
Oedema^a^	1.44 ± 0.86	1.88 ± 0.62	0.00 ± 0.00	0.00 ± 0.00
Neutrophil infiltration^a^	1.49 ± 1.20	1.72 ± 1.58	0.40 ± 0.54	0.00 ± 0.00
Haemorrhage	0.16 ± 0.28	0.27 ± 0.68	0.60 ± 0.54	0.40 ± 0.54
Epithelial desquamation	0.11 ± 0.17	0.00 ± 0.00	0.00 ± 0.00	0.00 ± 0.00
Hyaline membrane^b^	0.05 ± 0.13	0.85 ± 0.69	0.00 ± 0.00	0.00 ± 0.00

Total score^a^	3.48 ± 2.37	4.41 ± 1.96	1.00 ± 1.00	0.40 ± 0.54

Values are mean ± SD. P-NtV: pigs with normal tidal volume; P-HtV: pigs with high tidal volume; Rat-NtV: rats with normal tidal volume; Rat-HtV: rats with high tidal volume.

^a^ *P* < 0.05 between pig and rat groups.

^b^ *P* < 0.05 between P-HtV and P-NtV groups.

**Table 2:** Respiratory parameters and wet/dry weight ratio.

	Pig groups		Rat groups	
	P-NtV	P-HtV	Rat-NtV	Rat-HtV
Tidal volume (mL)^a, b^	270.0 ± 31.6	1583.3 ± 51.6	3.7 ± 0.3	15.8 ± 1.7
Plateau airway pressures (cm H_2_O)^a, b^	13.5 ± 1.7	57.3 ± 8.2	12.0 ± 1.1	38.3 ± 4.3
Wet/dry ratio^a, b^	4.33 ± 0.99	5.60 ± 0.37	4.43 ± 0.10	4.10 ± 0.07

Values are mean ± SD. P-NtV: pigs with normal tidal volume; P-HtV: pigs with high tidal volume; Rat-NtV: rats with normal tidal volume; Rat-HtV: rats with high tidal volume.

^a^ *P* < 0.01 between P-HtV and P-NtV groups.

^b^ *P* < 0.01 between Rat-HtV and Rat-NtV groups.

**Table 3:** Area of alveolar collapse in morphometric analysis.

	Pig groups		Rat groups	
	P-NtV	P-HtV	Rat-NtV	Rat-HtV
AAC, total^a, c^	43.1 ± 10.5	54.31 ± 8.4	38.5 ± 10.2	26.8 ± 4.9
AAC in upper lobes^b^	34.5 ± 7.2	46.3 ± 4.8		
AAC in medium lobes	45.8 ± 10.9	56.4 ± 8.6		
AAC in lower lobes^b^	48.8 ± 7.7	60.1 ± 3.8		

Values are percentages, mean ± SD. P-NtV: pigs with normal tidal volume; P-HtV: pigs with high tidal volume; Rat-NtV: rats with normal tidal volume; Rat-HtV: rats with high tidal volume; AAC: area of alveolar collapse.

^a^ *P* < 0.05 between pig and rat groups.

^b^ *P* < 0.05 between P-NtV and P-HtV groups.

^c^ *P* < 0.05 between Rat-NtV and Rat-HtV groups.
